# Evidence for recency of practice standards for regulated health practitioners in Australia: a systematic review

**DOI:** 10.1186/s12960-023-00794-9

**Published:** 2023-02-24

**Authors:** Penelope Ann Elizabeth Main, Sarah Anderson

**Affiliations:** 1 Australian Health Practitioner Regulation Agency, GPO Box 9958, Melbourne, VIC 3000 Australia; 2 La Trobe University, Bundoora, VIC Australia

**Keywords:** Recency of practice, Skill retention, Health practitioners, Regulatory standards, Systematic review

## Abstract

**Background:**

Health practitioner regulators throughout the world use registration standards to define the requirements health practitioners need to meet for registration. These standards commonly include recency of practice (ROP) standards designed to ensure that registrants have sufficient recent practice in the scope in which they intend to work to practise safely. As the ROP registration standards for most National Boards are currently under review, it is timely that an appraisal of current evidence be carried out.

**Methods:**

A systematic review was conducted using databases (including MEDLINE, EMBASE, PsycInfo, and CINAHL), search engines, and a review of grey literature published between 2015 and April 2022. Publications included in the review were assessed against the relevant CASP checklist for quantitative studies and the Joanna Briggs Institute checklist for analytical cross-sectional studies.

**Results:**

The search yielded 65 abstracts of which 12 full-text articles met the inclusion criteria. Factors that appear to influence skills retention include the length of time away from practice, level of previous professional experience and age, as well as the complexity of the intervention. The review was unable to find a clear consensus on the period of elapsed time after which a competency assessment should be completed.

**Conclusions:**

Factors that need to be taken into consideration in developing ROP standards include length of time away from practice, previous experience, age and the complexity of the intervention, however, there is a need for further research in this area.

**Supplementary Information:**

The online version contains supplementary material available at 10.1186/s12960-023-00794-9.

## Background

Health practitioner regulators in Australia and other jurisdictions define the requirements that applicants and registrants need to meet to become or stay registered. These standards are an important part of the regulatory framework for each profession and commonly include standards for primary education in the profession, continuing professional development (CPD), and recency of practice (ROP).

In 2010, Australia introduced the National Registration and Accreditation Scheme (the National Scheme) which regulates 16 health professions under the Health Practitioner Regulation National Law (the National Law). The National Law requires that National Boards must develop, consult on, and recommend certain registration standards to the Ministerial Council. ROP requirements aim to ensure that a health practitioner has sufficient recent practice in the scope in which they intend to work and that they have maintained an adequate connection with their profession to ensure they can practise competently and safely [1]. Health practitioners can become clinically inactive for a range of reasons including caring for family members, career dissatisfaction, health-related absences, the pursuit of other careers (including leadership and academic roles) [2] and the participation in approved research training or other educational opportunities [3]. In some cases, this may take the form of a career break, whereas other practitioners may be clinically active but are doing a low volume of work or be seeking to change their scope of practice.

The core registration standards are generally reviewed by National Boards every five years in line with good regulatory practice. Previous reviews of ROP standards were underpinned by two unpublished systematic reviews designed for internal use. These were a commissioned systematic review conducted by Professor Elizabeth Farmer in 2012 and an update of that review by the Australian Health Practitioner Regulation Agency (Ahpra) in 2015. Both concluded that ROP has been a poorly researched area with little known about the potential effect on the competence of practitioners who are re-entering the workforce, the best way to maintain competence during career breaks and the optimal way to assess the competence of returning practitioners. Furthermore, neither was able to find any clear consensus about the optimal time period after which assessment of competence should be introduced [4–6]. As the ROP registration standards for most National Boards are currently under review, it is timely to review the current evidence.

### Aim

The aim of the systematic review is to develop a contemporary evidence base to support the development of effective ROP registration standards.

### Research questions

1. Does the period of time for skills-retention and/or skills-fade vary between different health professions and/or at different stages of their career (e.g., new graduate, early career, mature or advanced practitioners)?
2. Is there evidence regarding when competency assessment should be completed?
3. Is there any evidence for the minimum number of hours of practice needed over a set period of time to maintain competency? Does this vary across professions or scope of practice?

## Method

A systematic review was conducted examining the above research questions based on selection criteria, methods and analysis that are summarised below.

The development of the research questions and search terms was informed by the two unpublished reviews discussed above.

The full protocol for the systematic review was recently published [7].

### Searching and screening

The search terms and sources of literature selected for the review are based on Ahpra’s previous experience conducting a systematic review of the evidence for ROP standards, which covered journal articles and grey literature published between 1990 and 2014, as well as standard database preliminary testing.

#### Search terms

Search terms were selected for the health practitioner group, intervention, and outcome using a combination of the National Library of Medicine Medical Subject Headings (MeSH) and additional relevant search terms. Boolean operators were used to combine terms, and ‘wild cards’ were used to account for plurals and variations in spelling. MeSH is a standardised hierarchically organised vocabulary developed by the National Library of Medicine to index, catalogue and search biomedical and health related information. The MeSH terms for this review are presented in an Additional file 1: Appendix A and can also be found in the published protocol [7].

#### Sources of literature

The main sources of literature were:

- *Research databases* including the Medical Literature Analysis and Retrieval System On-line (MEDLINE), Excerpta Medica dataBASE (EMBASE), and PsycINFO (using the OVID platform) and the Cumulative Index of Nursing and Allied Health Literature (CINAHL)
- *Search engines* comprising Google Scholar and Google Advanced
- *Grey literature* produced by other regulatory organisations, governmental bodies and professional associations.
- *Reference lists* of papers and reports selected for review.

#### Inclusion and exclusion criteria

Articles and reports were included in the systematic review if they met the following criteria:

1. the focus of the report/article was ROP for those health professions regulated in Australia
2. reviews, original research, reports and theses
3. published from 1 January 2015 to mid April 2022
4. written in the English language

Articles and reports were excluded from the systematic review if they met the following criteria:

1. focussed on health and other professionals not regulated under the National Law
2. focussed on students or interns
3. focussed on regulatory standards other than ROP
4. opinion pieces, newsletters, conference presentations
5. published before 1 January 2015
6. not written in the English language.

#### Data extraction

Titles identified from the search were checked and the abstract was reviewed where the title appeared to be relevant to the research questions. Where the abstract met the inclusion criteria the full article was downloaded and assessed against the inclusion/exclusion criteria.

A Microsoft Excel spreadsheet was used to record bibliographic information about each article or report (e.g., author, date, title), the study population (e.g., health profession, size, country), intervention (e.g., return to practice, maternity/paternity leave), main findings, study type, the Australian National Health and Medical Research Council (NHMRC) level of evidence [8], decisions as to inclusion/exclusion (including any reasons for exclusion) and the quality assessment.

### Quality appraisal

Where the full text of the article was assessed as relevant to the research question(s), a quality appraisal was conducted independently by two people. The protocol was modified to use the Critical Appraisal Skills Programme (CASP) checklists for systematic reviews [9], randomised controlled trials and cohort studies for quality appraisal of our yield. Items on the CASP checklist for systematic reviews were left out where they were not deemed relevant for a narrative review. The Joanna Briggs Institute (JBI) checklist for appraisal of analytical cross sectional studies was also used [10].

## Results

### Study selection

**Fig. 1:**
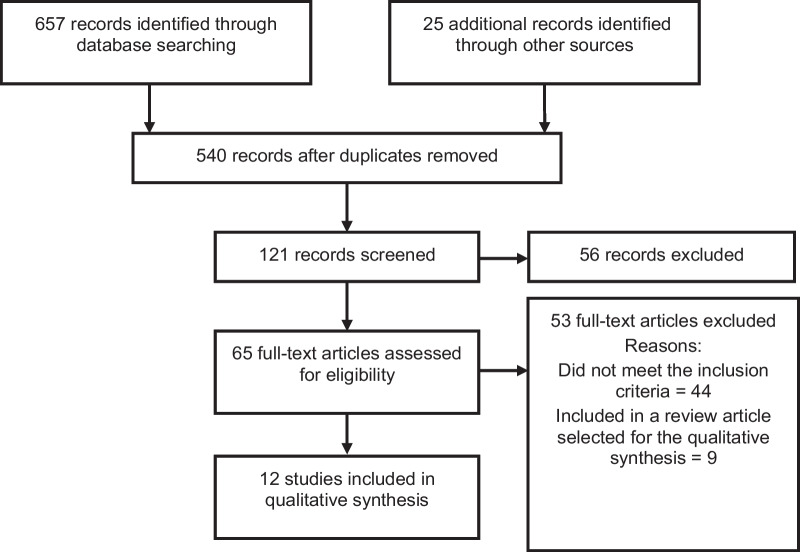
Flow chart of studies selected for inclusion in the systematic review

Our search strategy identified 657 studies through database searching, with an additional 25 records identified through other sources, resulting in 540 records after duplicates were removed. Of these, 121 records were screened based on their title and 56 records were excluded. Sixty-five full text articles were assessed for eligibility based on their abstract, of which 53 full-text articles were excluded. Reasons for exclusion were either because they did not meet the inclusion criteria (44) or they were included in a review article selected for the qualitative synthesis [9] (Fig. 1).

### Description of included studies

Twelve studies were included in the review; comprising five literature reviews (two systematic reviews, three narratives), a randomised controlled trial and five cohort studies which were assessed for quality using the relevant CASP checklist. There was also a cross-sectional study that was assessed using the relevant JBI checklist. The characteristics and quality assessment of the included studies are outlined in Table 1.

**Table 1:** Characteristics and quality assessment of the included studies

Study design	Author	Country	Participants	Study size	Relevant research questions	Quality
Systematic review	Campbell et al*.* 2018	Scotland	Occupational therapists, paramedics, physiotherapists, podiatrists, psychologists, medical radiation practitioners	39 studies	RQ 1 – skills fade	Moderate
	Thim et al*.* 2022	Denmark	Nurses, medical practitioners, midwives	8 studies	RQ 1 – skills fade	Moderate
Narrative review	Atesok et al*.* 2016	United States	Orthopaedic residents	21 studies	RQ 1 – skills fade	Moderate
	Gawad et al*.* 2019	Canada	Surgical residents	5 cohort studies	RQ 1 – skills fade RQ 2 – competency assessment	Moderate
	Maddocks et al*.* 2020	New Zealand	Military general practitioners, ICU/ emergency nurses, military and civilian nurses, resident medical officers	10 studies	RQ 1 – skills fade RQ 2 – competency assessment	Moderate
Randomised Controlled Trial	Jani et al*.* 2019	United States	Paediatric residents	Intervention arm *N* = 12 Control *N* = 12	RQ 1 – skills fade	Low
Cohort study	Clark et al*.* 2022	United States	Academic anaesthesiologists	*N* = 61 participants	RQ 1 – skills fade	Moderate
	Harkemanne et al*.* 2021	Belgium	General practitioners	*N* = 89 participants	RQ 1 – skills fade	Low
	Nathwani et al*.* 2017	United States	Surgical residents	*N* = 27 participants	RQ 1 – skills fade	Moderate
	Paliatsiou et al*.* 2021	Greece	Paediatricians, anaesthesiologists, paediatric residents, midwives, nurses and paramedics	*N* = 116 participants	RQ 1 – skills fade	Moderate
	Schott et al*.* 2021	United States	Medical practitioners	*N* = 127 participants	RQ 1 – skills fade	Moderate
Cross-sectional study	Maubon et al*.* 2021	United Kingdom	Ophthalmic surgeons	*N* = 232 participants	RQ 1 – skills fade	Moderate

The two systematic reviews were assessed as of moderate quality, however, the focus of those reviews have limited applicability to the research questions [11, 12]. The three narrative reviews included in our study were rated as of moderate quality [13–15]. The findings of these reviews should be treated with caution as the number of participants in each of the included studies were low and follow-up times were short.

The randomised controlled trial was assessed as low quality because the number of participants in each arm (*n* = 12) was too small to show an effect size and participants in the intervention group had significantly more previous experience than those in the control arm [16].

Four cohort studies were assessed as of moderate quality [17–20] and the fifth was of low quality [21]. The main limitations of the first medium quality study were that 25% of study participants were lost to follow-up and the analysis did not consider the length of previous experience in the area of training [17]. The main limitation for the second study was that it was a small study that used a pre-test, post-test design to assess skill retention in simulated surgical activities [18]. The third study followed up almost all participants to assess the retention of their knowledge, but retention of technical skills was only assessed in 69% of participants, the time for follow-up was relatively short (3 and 6 months) and potential sources of bias are not discussed [19]. The fourth study did not include an analysis of the findings by previous experience with the point-of-care ultrasound techniques that were the subject of the study [20]. Limitations of the low quality cohort study included insufficient consideration of potential sources of bias or confounding and high loss to follow-up [21].

The cross-sectional study was assessed as of moderate quality, its main limitation being a reliance on self-reported issues rather than objective structured testing of performance after a break of at least eight months due to the COVID-19 pandemic [22].

### Research question 1

*Does the period of time for skills-retention and/or skills-fade vary between different health professions and/or at different stages of their career (e.g., new graduate, early career, mature or advanced practitioners)?*

#### Variation in skills retention and/or skills fade between different health professions

The review identified only one study that compared skill retention or skills fade between different health professions [19]. It found that, following completion of a course on neonatal life-support, medical practitioners (paediatricians, anaesthetists and residents) (*N* = 74) had significantly higher retention of theoretical knowledge and technical skill compared to other health practitioners (midwives and other unspecified health practitioners) working in a neonatal setting (*N* = 40), at baseline, 3 and 6 months.

We found that almost all the published literature about skills fade focussed on medical practitioners, nurses or paramedics. The literature generally concentrated on specific skills associated with a higher risk to public safety, such as surgical or resuscitation procedures, for which the participants were generally health practitioners in active practice. While some of the studies examining skills fade in a pre-hospital setting comprised a mixture of health professions, none of the other authors stratified their findings by profession.

The review identified two narrative reviews, a randomised controlled trial and five cohort studies that examine skills decay in active practitioners following training that are of medium to low quality [13–21]. In addition, a highly relevant systematic review of skills fade conducted by the General Medical Council (GMC) in 2014 is cited by a systematic literature review identified in this study [11].

The GMC’s systematic review of skills fade concluded that clinical skills decline between six and 18 months, with a steeper decline at the outset and a more gradual decline as time passes [4].

In reaching its conclusions, the GMC review noted older empirical research indicates that:

- speed/time to complete a task and accuracy are commonly used as main outcome variables to assess skill decay
- the extent of skills fade is primarily largely determined by the degree of over-training and the complexity of the task [23].

#### Skills decay in active practitioners

The review found that most studies of skills decay are based on studies of novice or recent graduate health practitioners. Although the subject of this review is ROP of practitioners returning to the workforce, studies examining skill decay in active practitioners can provide useful information, particularly where the practitioner did not use the skill of interest during the lapsed time. Skills retention in active health practitioners varies with the task. For instance emergency airways management and defibrillation skills decrease between four and six months after training [4, 12, 15, 19], whereas laparoscopic surgical skills decrease six to eight months after training, and catheter insertion skills for haemodialysis do not decrease until after one year [4]. Longer breaks were generally associated with greater skill decay [14].

Evidence for the extent of skill decay was inconsistent, with one review concluding that practitioners assessed for retention of learned skills did not lose the skill completely when tested between four months and two years after training [4] while another reported a complete loss of skills in orthopaedic residents who had trained in simulation-based arthroscopic shoulder skills and not used the skills after six months [13]. Another reported an 80% retention rate of airway skills learned by anaesthetists who had undergone simulation emergency airway training [17].

Regular repeated assessments of basic surgical skills during surgical residents’ research years improved errors associated with rule-based procedures, but did not improve errors associated with the strategic approach to the surgical intervention compared to residents returning to surgery who had not undergone repeated assessment [18]. Residents and faculty perceive that the extent of skills decay is related to the level of skill difficulty, with the greatest loss perceived in technical skills, followed by a decrease in knowledge of procedural steps [4, 14].

Practice between assessments was reported to increase confidence and proficiency, with experts appearing to retain skills better than novices [4, 13–15]. For example, orthopaedic residents who had attended a 30-day intensive course that included basic fracture fixation techniques, application of casts and splints, and familiarisation with basic surgical instruments had significantly greater skill retention than non-participants after six months [13]. Similarly, residents who were exposed to surgical skills through on-call work during a research break from a clinical role of one to three years reported higher confidence to undertake more difficult surgical procedures at the completion of the research absence compared to surgical residents whose on-call experiences were limited to bedside care [14]. Retention of paediatric resuscitation skills eight months after training was improved by additional simulation-based training at four months [16].

The role of practice between assessments is also reflected in a study that showed a significant increase in the use of point-of-care ultrasound by active medical practitioners following completion of a continuing medical education course which was associated with increased skills retention at eight months [20]. Knowledge test scores increased from a median of 60% to 90% immediately post-course, which decreased to 87% eight months after the course. Median skills test scores for four common applications (heart, lung, abdomen and vascular access) increased from 36 to 72 points immediately post-course with a two-to-seven-point decrease after eight months. Pre-course knowledge and skills test scores were significantly lower for non-users compared to moderate-to-high users, however, this discrepancy was diminished immediately post-course and retained after eight months.

This review also identified a poorly designed study of skill retention in general practitioners following a one hour training session in melanoma diagnosis and treatment which found that although there was a significant increase in knowledge immediately after training, the 30% of participants followed up at the end of a year had significantly lower scores for appropriate management of cases compared to immediately after training [21].

#### Skills decay after time away from practise

A single study specifically examined the impact of skill decay after time away from practise used the subjective experiences of United Kingdom ophthalmologists returning to cataract surgery after a nationwide pause on elective surgery of eight months due to the COVID-19 pandemic [22]. This study found that two-thirds of respondents were unaware of any return to practice guidelines and only one in nine respondents had a formal plan made before returning to cataract surgery. Operating difficulties were frequently reported after returning to cataract surgery (29.1%), particularly by less experienced ophthalmic surgeons.

### Variation in skills retention and/or skills fade between different stages of a health practitioner’s career

The strongest empirical evidence for variation in skills retention and/or skills fade between different stages of a health practitioner’s career comes from a study of medical practitioners returning to practice in the United States [2] which was published prior to the study period for this review. The study found that older age and longer time out of practice are significantly related to performance at assessment for a return to practice program. Assessment of skills at the time of re-entry showed that only a quarter of participants (15, 24%) were competent to return to practice with no or minimal education, while more than two-thirds (43, 69.4%) required remediation through a structured educational process and a small proportion (4, 6.5%) were assessed as requiring training in a residency program. Linear regression demonstrated that years out of practice and increasing physician age predicted poorer performance.

The only comparison of skill retention in novices and experts identified in the review found that experienced surgeons demonstrated a high degree of skills retention 18 months after training in laparoscopic procedures, whereas novices provided with the same training returned to the pre-training level between six and 18 months afterwards [4].

### Research question 2

*Is there evidence regarding when competency assessment should be completed?*

There is no clear consensus on the period of elapsed time after which an assessment of competency may be needed. The findings indicate that the need for a competency assessment after an absence from practice depends on the skills and circumstances of the individual health practitioner [4, 14, 15, 24].

The GMC review of skills fade found no consensus on the period of elapsed time after which an assessment of competence should be introduced concluding that, based on the evidence it had collected, when a competency assessment should be completed depends on the skill and the circumstances of initial acquisition and interim practice [4]. Gawad et al. 2019 reached a similar conclusion, noting that surgical residents who were returning to clinical training after an extended period of research training were treated as if their clinical training had not been interrupted [14].

The systematic review of skills fade of emergency airway management found that none of the 10 studies (which covered hospital, military deployed and domestic settings) included in the review were able to recommend an ideal time for refreshing [15]. However, a Delphi study of military nurses (included in that review) found strong agreement among experts that a return to practice program should be required following a period of 18 months out of clinical practice, with a very strong agreement for nurses returning after two years [24], suggesting a need for competence assessment around this time.

### Research question 3

*Is there any evidence for the minimum number of hours of practice needed over a set period of time to maintain competency? Does this vary across professions or scope of practice?*

The review identified only one reference to objective evidence for a minimum number of hours of practice to maintain competency. This was to the Texas Board of Nursing’s adoption of a four-year threshold for nurses returning to practice or transitioning to a new practice setting which is based on joint research conducted by the Texas Board of Nursing and Lamar University in 1994 that showed an increased risk of errors leading to disciplinary action in nurses returning to practice after more than four years [25]. Unfortunately, this research is unpublished.

## Discussion and conclusions

Australian and international health practitioner regulators have specific requirements to ensure their registrants practise safely and professionally. In Australia, these requirements include a minimum duration of practice, maximum time away from practice, and maximum time between completing a qualification and starting practice. Health practitioners can become clinically inactive for a range of reasons including caring for family members, career dissatisfaction, health-related absences, the pursuit of other careers (including leadership and academic roles) [2] and the participation in approved research training or other educational opportunities [3]. Common concerns reported by health practitioners returning to work include anxieties about loss of clinical skills and knowledge, low self-confidence, work–life balance, and fears about how they will be perceived by colleagues [3, 26–28].

A systematic review conducted by Ahpra in 2015 (unpublished) of the evidence for ROP which focussed on Aboriginal and Torres Strait Islander health practitioners, Chinese medicine practitioners, medical radiation practitioners and occupational therapists found almost all studies were of medical practitioners, nurses or midwives. That review concluded that there was very little evidence about the amount of recent practice required to maintain competence, although the findings of a small study suggest that shorter breaks from practice (one to five years) have less impact on competence than longer breaks (more than five years) [2].

This review, which covers all health professions regulated under the National Scheme, found a small body of research on ROP published in the last five years. As for the previous review, almost all studies were of medical practitioners, nurses and midwives. Aside from three large literature reviews, these studies generally focus on specific areas of practice that require a high level of clinical skill and accuracy. In short, higher risk areas of practice, such as surgery, pre-hospital emergency medicine or military deployment.

### Skills retention or fade

This review found there was consistent evidence that skills for high-risk procedures decline between six and 18 months after training, and resuscitation skills after four and 12 months. While the evidence is limited, its implications may be particularly important for health practitioners who have a low volume of cases requiring more complex skills, such as those who work on a casual basis and/or less than full time.

Our findings support those of the GMC 2014 review of skills fade which found that clinical skills decline over periods ranging from six to 18 months out of practice, with a steeper decline at the outset slowing to a more gradual decline as time passes, and for resuscitation skills, the decline appears to occur between four and 12 months after training [4].

Are these findings applicable to health professionals who carry out lower risk procedures? Our review found little research on ROP for other health professions. Future research may be warranted to develop an understanding of the risks of those returning to practice following a period of absence. Only one of the studies identified through this review directly compared skills retention or fade across professional groups [19]. This study showed that medical practitioners had better skills retention than other health professionals (midwives, nurses and paramedics) following training in paediatric resuscitation skills. While the studies of skills fade in surgical residents and the military medical corps focussed on individual professional groups, some of the older studies of skills fade following resuscitation training cited by the GMC’s review included mixed professions, generally medical practitioners and nurses, also including midwives and paramedics [4]. The authors found no difference in skills fade between health professions following training, the critical factors appearing to be the complexity of the task and repetition [29–31].

This review identified only one study that compared experts with novices [32]. It found that there was higher skill retention for laparoscopic procedures in experts than novices. Other studies found an association between greater skills fade and older age, longer time out of practice, and a lower volume of relevant cases [2, 33, 34]. Analyses of notifications to Ahpra have consistently found that older age is associated with an increased risk of notification. The inter-relationship between older age and poorer skills retention is likely to be complex. Training in procedures that involve fine motor skills was shown to decline relatively quickly [35] and poorer cognitive skills are also likely to play a part [36].

### Competency assessment

How can regulators assess the competency of health practitioners who do not meet ROP standards? A literature review on best practice in the assessment of the competency of medical practitioners published in 2018 found that regulators need to be clear about what construct they may wish to assess [37] as the design will depend on whether they are interested in global judgements, specific behaviours, or the ability to demonstrate a professional response. While ethical challenges in medicine are universal, expected standards of performance may vary with the level of training and practice, requiring flexibility in the approach for assessment. Assessment of patient safety should centre on the candidate’s understanding of safety as a process, rather than technical competence.

### Minimum number of hours over set time to maintain competency

The review was unable to find any evidence of the minimum number of hours required to ensure that competency is maintained other than a reference to an unpublished 1994 study of Texas nurses [25]. Research using regulatory data is needed to determine whether there is an association between time out of practice and risk of complaints, particularly those leading to disciplinary action and the optimal time for return to practice. As well as providing an objective foundation for the protection of the public, the findings could potentially also lead to less variation between jurisdictions.

### Areas for further research

The review identified a number of areas where research could strengthen the evidence base for ROP standards. Suggested research includes benchmarking regulatory standards across jurisdictions and health professions, a case–control study to examine the risk of performance related complaints about health practitioners returning to work after varying times out of the workforce (e.g., one, three and five years), and extension of the systematic review to include self-regulated health professions and other regulated professions (e.g., teachers, lawyers).

### Limitations

The main limitation of this review is the paucity of high-quality relevant studies. Almost all the research has been carried out on medical practitioners, nurses or midwives, and none of the publications compare ROP across professions. The bulk of the research centres on skills fade for high-risk procedures rather than ROP per se. Generally, these studies have low numbers of participants (except some of the studies of skills retention following resuscitation training), relatively short follow-up times, and some of the studies of skills fade in active practitioners do not make clear the extent of practice between training and assessment. Another limitation is the exclusion of self-regulated health professions and other regulated professions (e.g., teaching, law). The findings of this review should, therefore, be treated with caution.

### Conclusions

ROP continues to be an under-researched area with most studies focusing on medical practitioners, nurses and paramedics in active practice. Studies of skills retention following training by novices focus exclusively on complex procedures such as resuscitation skills or surgical techniques. Although the exact nature of the inter-relationship between them is unknown, factors that appear to influence skills retention include the length of time away from practice, level of previous professional experience and age. With the exception of paramedics, surgeons and health practitioners in a military environment, complex high-risk procedures tend to be the exception in most practice situations, which should be reflected in ROP requirements. It is not known if the findings generalise to less-complex aspects of practice. Based on the available evidence, there is a case for ROP requirements that allow practitioners to undertake less-complex aspects of practice when they first return to work, before taking on the more complex aspects of practice following on-the-job assessment. This approach would assist regulators to balance the need to ensure patient safety with minimal impact on workforce supply.

The review was unable to find either a clear consensus on the period of elapsed time after which a competency assessment should be completed or any objective evidence for the minimum number of hours practice over a set period of time needed to maintain competency, although the individual skill and circumstances of the individual health practitioner appear to be important factors. There is a need for further research based on regulatory data to ensure that regulatory requirements for ROP are based on the best available evidence.

## Supplementary Information

**Additional file 1:** Appendix A.

## Data Availability

The data and material are listed in the reference list.
