# Supplementary material for: Evidence for recency of practice standards for regulated health practitioners in Australia: a systematic review
Source: Hum Resour Health. 2023 Feb 24;21:14. doi: 10.1186/s12960-023-00794-9 (PMC9951142; doi:10.1186/s12960-023-00794-9)
Supplement: Supplementary file 1 — Additional file 1: Appendix A. [file 12960_2023_794_MOESM1_ESM.docx]

**Appendix A**

| **MEDLINE** |  |
| --- | --- |
|  | Exp Health personnel/ OR health care profession*.ti,ab OR Chinese adj2 herbal*,ti,ab OR ((Chinese med*)adj2 prac*) OR chiropract*.ti,ab OR dent*.ti,ab OR doctor*.ti,ab OR general practitioner*.ti,ab OR ((Exp health workforce/ AND Indigenous AND Australia) OR medica*.ti,ab OR midwif*.ti,ab OR nurs*.ti,ab OR occupational therap*.ti,ab OR optom*.ti,ab OR osteopath*.ti,ab OR paramedic*.ti,ab OR pharm*.ti,ab OR physician*.ti,ab OR physiotherap*.ti,ab OR physical therapist*.ti,ab OR podiatr*.ti,ab OR psycholo*.ti,ab OR radiograph*.ti,ab OR early adj2 career OR mid adj2 career OR late adj2 career |
| AND | recency of adj2 pract*,ti,ab OR re-entry dj2 program OR refresher adj2 program OR registration adj2 requirement*,ti,ab OR Exp return to work/ |
| AND | career mobil*.ti.ab OR fitness to pract*.ti,ab OR Exp guidelines as topic/ OR Exp licensure/ OR Exp malpractice/ OR Exp mandatory programs/ OR Exp organizational policy/ OR Exp patient safety/ OR problem adj2 behavi* OR Exp professional competence/ OR professional adj2 pract* OR Exp professionalism/ OR Exp quality of health care/ OR regulat*, ti,ab OR Exp risk management/ OR scope adj3 of pract* OR exp social control, formal/ OR skills adj2 decay OR skills adj2 fade OR standards of practi*.ti,ab OR standard setting,ti,ab OR (standard*adj2 practi*),ti,ab |
| AND | Limit to yr=2015– May 2022 |
| AND | Limit to English language |
| **Embase** |  |
|  | Exp Health personnel/ OR health care profession*.tw OR Chinese med* adj2 prac* OR chiropract*.tw OR dent*.tw OR doctor*.tw OR general practitioner*.tw OR (Exp health workforce/ AND Indigenous.mp AND Australia.mp) OR medica*.tw OR midwif*.tw OR nurs*.tw OR occupational therap*.tw OR optom*.tw OR osteopath*.tw OR paramedic*.tw OR pharm*.tw OR physician*.tw OR physiotherap*.tw OR physical therapist*.tw OR podiatr*.tw OR psycholo*.tw OR radiograph*.tw OR early adj2 career OR mid adj2 career OR late adj2 career |
| AND | recency of adj2 pract*,tw OR re-entry adj2 program.tw OR refresher adj2 program OR registration adj2 requirement*.tw OR Exp return to work/ |
| AND | career mobil*.tw OR fitness to pract*.tw OR Exp guidelines as topic/ OR Exp licensure/ OR Exp malpractice/ OR Exp mandatory programs/ OR Exp organizational policy/ OR Exp patient safety/ OR problem adj2 behavi* OR Exp professional competence/ OR professional adj2 pract* OR Exp professionalism/ OR Exp quality of health care/ OR regulat*.tw OR Exp risk management/ OR scope adj3 of pract*.mp OR Exp social control/ OR skills adj2 decay.mp OR skills adj2 fade.mp OR standards of practi*, tw OR standard setting,mp OR (standard*adj2 practi*),tw |
| AND | Exp guidelines as topic/ OR Exp licensure/ OR Exp malpractice/ OR Exp mandatory programs/ OR Exp organizational policy/ OR Exp patient safety/problem adj2 behavi* OR Exp professional competence/professional adj2 pract* OR Exp professionalism/ OR Exp quality of health care/regulat*.tw OR Exp risk management/scope adj3 of pract*.mp OR Exp social control/ skills adj2 decay.mp skills adj2 fade.mp OR standards of practi*. tw OR standard setting,mp OR (standard*adj2 practi*).tw |
| AND | Limit to yr=2015– May 2022 |
| AND | Limit to English language |
| **PsycInfo** |  |
|  | Exp Allied health personnel/ OR Exp Health care profession/ OR Exp Health personnel/ OR Exp Acupuncturist/ OR Exp Dentists/ OR (Exp Health care services AND Exp Indigenous populations/ AND Australia) Exp Medical personnel/ OR Exp Midwifery/ OR Exp Military medical personnel/ OR Exp Nurses/ OR Exp Occupational Therapists/ OR Exp Optometrists/ OR Exp Osteopathic Medicine/ OR Exp Paramedical personnel/ OR Exp Pharmacists/ OR Exp Physical therapists/ OR Exp Physicians/ OR Exp Podiatrist OR Exp Psychiatric hospital staff/ OR Exp Psychologists/ OR Exp Radiographer/ OR Chiropractor mp OR Chinese adj medicine practitioner.mp OR early adj2 career.mp OR mid adj2 career.mp OR late adj2 career.mp |
| AND | recency of adj2 pract*,mp OR re-entry adj2 program.mp OR refresher adj2 program.mp OR registration adj2 requirement*.mp OR  Exp return to work/ |
| AND | Exp career mobility/ OR fitness adj2 pract*.mp OR Exp guidelines/ OR Exp licensing/ OR Exp malpractice/ OR mandatory adj2 programs.mp OR Exp organizational policy/ OR Exp patient safety/ OR problem adj2 behavi*.mp OR Exp professional competence/ OR professional adj2 pract*.mp OR professional adj2 regulat*.mp OR Exp professional standard/ OR Exp professionalism/ OR Exp quality of care/ OR Exp risk management/ OR  scope adj3 of pract*.mp OR Exp Social control/ OR skills adj2 decay.mp OR  skills adj2 fade.mp |
| AND | Limit to yr=2015– May 2022 |
| AND | Limit to English language |
| **CinAHL** |  |
|  | MH Health personnel OR MH Allied Health Personnel OR MH Physicians OR MH Nurses OR MH Registered nurses OR MH Midwifery OR MH Chiropractic OR MH Dentists OR MH Emergency Medical Technicians OR MH First National of Australia OR MH Occupational Therapists OR MH Osteopaths OR MH Optometrists OR MH Pharmacists OR MH Physical Therapists OR MH Podiatry OR MH Psychologists OR MH Radiologic Technologists OR (TI (Nurs* OR optom* OR dent* OR midwif* OR pharm* OR medica* OR osteopath* OR chiropract* OR podiatr* OR occupational therap* OR paramedic* OR physiotherap* OR psycholo* OR radiograph* OR doctor* OR physician* OR general practitioner* OR health care profession* OR healthcare profession* OR physical therapist* OR early n2 career OR mid n2 career OR late n2 career)) OR (AB ( Nurs* OR optom* OR dent* OR midwif* OR pharm* OR medica* OR osteopath* OR chiropract* OR podiatr* OR occupational therap* OR paramedic* OR physiotherap* OR psycholo* OR radiograph* OR doctor* OR physician* OR general practitioner* OR health care profession* OR healthcare profession* OR physical therapist* OR early n2 career OR mid n2 career OR late n2 career)) |
| AND | MH Job re-entry OR MH Refresher courses OR (TI (recency of n2 pract* OR registration n2 requirement*)) OR (AB (recency of n2 pract* OR registration n2 requirement*)) |
| AND | MH Career mobility OR MH Clinical competence OR MH Malpractice OR  MH Organizational policies OR MH Patient safety OR MH Professional competence OR MH Professional misconduct OR MH Professional practice OR MH Professional regulation OR MH Professionalism OR MH Quality of health care OR MH Risk management OR MH Skill retention OR MH Scope of practice OR (TI (fitness to practi* OR standard setting OR standard* n2 practi* OR mandatory n2 programs OR problem n2 behavi*)) OR (AB (fitness to practi* OR standard setting OR standard* n2 practi* OR mandatory n2 programs OR problem n2 behavi*)) |
| AND | Limit to yr=2015– May 2022 |
| AND | Limit to English language |
